# Effectiveness of Jacobson Relaxation and Lamaze Breathing Techniques in the Management of Pain and Stress During Labor: An Experimental Study

**DOI:** 10.7759/cureus.33212

**Published:** 2023-01-01

**Authors:** Gayatri S Kaple, Shubhangi Patil

**Affiliations:** 1 Department of Physiotherapy, Datta Meghe Institute of Medical Sciences, Wardha, IND

**Keywords:** normal vaginal delivery, perceived stress, numerical pain rating scale, lamaze breathing exercise, jacobson relaxation technique, stress, labor pain

## Abstract

Background and objective

The mother's ability to tolerate labor discomfort has an impact on how the labor progresses. Good pain management will boost mothers' ability to labor collaboratively and will shorten the time it takes for the uterus to open completely. Many women prefer not to use pharmaceutical or invasive pain relief during labor, which may have contributed to the popularity of complementary pain relief approaches. This study aimed to assess the effectiveness of the Jacobson relaxation technique and Lamaze breathing technique in the management of pain and stress during labor.

Methods

Thirty-six women aged between 25 and 35 years were randomly assigned to two groups for the purpose of this study. Group A received the Jacobson relaxation technique while Group B received the Lamaze breathing technique for four weeks. The patients were instructed to practice breathing techniques at the time of labor. The outcomes measure included the Numerical pain Rating Scale (NPRS) and Perceived Stress Scale (PSS), which would be measured before and after the delivery.

Results

The subjects showed improved labor pain and anxiety following the physiotherapy intervention. The results were found to be statistically significant (p<0.05).

Conclusion

Based on our findings, physiotherapy intervention plays an integral role in the multidisciplinary approach to relieving labor pain and helping patients have a normal vaginal delivery.

## Introduction

Labor pain is a physiological phenomenon caused by the smooth muscles of the uterus contracting to assist the fetus down the birth canal [1,2]. The process of the cervix opening and thinning and the fetus descending the birth canal causes pain during labor. There are many causes of this pain, such as experience, fear, and anxiety along with racial, cultural, social, and environmental factors, and demographic and biological characteristics [3]. Severe labor anxiety increases the likelihood of a cesarean section, which may be followed by cesarean delivery-related difficulties for both mother and fetus, putting financial strains on the family and the state and lengthening hospitalization time [4]. Various pharmacological and non-pharmacological techniques for regulating and lowering labor pain have been proposed to date [5]. One of these approaches that have received increased attention from researchers is relaxation techniques [6]. The relaxation technique is a cognitive behavioral approach that is used to control emotions. This technique can divert a person’s mind from stress, fear, anger, and discomfort [7]. The Jacobson relaxation technique focuses on the specific area of tensing and relaxing the muscles and is also known as progressive relaxation treatment [8]. Jacobson proposed that tensing and then relaxing a muscle relaxes it further. He also suggested that it is possible to calm the mind by doing so. A framework for obtaining this level of relaxation is provided by progressive muscle relaxation. Relaxation methods are employed as coping strategies during labor to relieve pain by disrupting sensations of pain, restricting the ability to pay attention to the perception of pain, promoting the production of endorphins, and assisting in the reduction of pain-exacerbating thoughts [9].

The term "breathing technique" has long been associated with the Lamaze method of childbirth education. It is considered that the breathing technique is successful because it serves as a diversion, drawing attention away from the discomfort [10]. There are many breathing patterns to choose from and rigid rules for "doing it correctly." The combination of breathing and relaxation techniques can lower pain perception to the point that women could give delivery without needing medicines. Lamaze breathing is a breathing method that is based on the concept that regulated breathing can help patients feel relaxed and reduce pain. Lamaze is a comprehensive program that inspires confidence and keeps things simple for a safe and healthy birth. Changing postures, walking, slowly swaying, and massage are among the labor comfort strategies prescribed to make breathing techniques more effective [11]. In light of this, this study aims to analyze the effectiveness of Jacobson’s relaxation and Lamaze breathing techniques in the management of pain and stress during labor. We hypothesized that the Jacobson relaxation and Lamaze breathing techniques would be equally effective in relieving pain and stress during labor. Physiotherapy may be effective for a pregnant woman during her pregnancy, assisting in the prevention of common pregnancy-related discomforts. The Jacobson relaxation technique and Lamaze breathing assist in reducing labor pain and anxiety [12].

There are three phases of labor [13], which are as follows - first stage: the contractions that force the cervix to open begin at this point and continue until the cervix has dilated fully; second stage: the cervix has fully dilated at this phase, and the woman would continue pushing the baby out; third stage: this occurs after the infant is delivered and the placenta is expelled. Jacobson’s relaxation technique and Lamaze breathing exercises are provided during the first stage of labor.

The Jacobson relaxation technique is a kind of therapy in which certain body muscle groups from toe to head are involved to achieve overall body relaxation. Focusing on specific areas and tensing and releasing them is known as progressive relaxation treatment [14]. It is essential to focus on one muscle group at a time. This enables the patient to recognize the stress in that particular place. Before relaxing, it is also important to tense the body muscle group. This activity enhances the area's relaxing experience [15]. This technique is beneficial in many ways as it reduces anxiety and low back pain and improves systolic blood pressure [16].

Relaxation methods are employed as coping strategies during labor to relieve pain by disrupting sensations of pain, restricting the ability to pay attention to the perception of pain, promoting the production of endorphins, and assisting in the reduction of pain-exacerbating thoughts [17]. In the past few years, Lamaze sessions have become very popular among couples and these involve practicing controlled breathing and conscious relaxation both in and out of class to prepare for labor [18].

Lamaze breathing is widely practiced today, and it is a method that is based on the concept that regulated breathing can help patients feel relaxed and reduce pain. Slow, deep breathing, by sustaining a constant rhythm, breathing from the mouth or nostrils, keeping the eyes open or closed, and concentrating on a single physical object, such as a photograph or your spouse, are all crucial tactics for regulated breathing. Those who advocate for the use of Lamaze claim that breathing is only one aspect of the Lamaze approach. Lamaze is a comprehensive program that instills expectant mothers with confidence and keeps things simple for a safe and healthy birth [19].

Several studies have compared different maternal positions in different stages of labor. However, there is scarce research focusing on the association of the Jacobson relaxation technique and Lamaze breathing with easing labor pain and stress. Hence, the present study intends to analyze the effectiveness of the Jacobson relaxation technique and Lamaze breathing in managing labor pain and stress.

## Materials and methods

Materials

The items utilized in the study were as follows: a printed copy of the data collection sheet, consent form, Numerical Pain Rating Scale (NPRS), Perceived Stress Scale (PSS), couch, and pillows. The methodology of the research design was the interventional mode. The study was designed as experimental research. The study setting was the Department of Community Health Physiotherapy, Ravi Nair Physiotherapy College, Acharya Vinoba Bhave Rural Hospital, Sawangi (Meghe), Wardha. The population studied was pregnant women. The sampling technique employed was the simple sampling technique, and the sampling method used was the envelope. The sample size was 35 patients classified into two groups. Group A included 18 subjects who were given the Jacobson relaxation technique, while Group B involved 18 subjects who were given the Lamaze breathing technique. The study duration was six months. The inclusion criteria were as follows: age between 25 and 35 years; primiparous women with a gestational age between 32 and 34 weeks; absence of complications throughout the pregnancy, and planned for normal delivery. The exclusion criteria were as follows: women with diabetes and hypertension, any other complications, and planned C-sections.

Parameters/Outcome Measures

NPRS: this scale is an 11-point scale on which patients assess the severity of their present pain from 0 ("no pain") to 10 ("worst agony possible"). It is used to indicate the current pain intensity as well as the best and worst levels experienced over the past 24 hours. The scale has been shown to have adequate reliability and validity.

PSS: a more precise measurement of personal stress can be determined using a range of tools developed to assess individual stress levels. PSS is the foremost among them. The PSS scores range from 0 to 40 points, with higher values indicating more stress.

Data Source Measurement

NPRS: it is reliable if r=0.96, and valid if correlations range from 0.86 to 0.95 [20].

PSS: the parameters of validity were r=0.32 and p<0.001 [21,22].

Procedures

The patients were administered the Jacobson relaxation technique and Lamaze breathing exercise. These exercises were taught throughout the pregnancy period so that the patient could perform them in the first stage of labor based on the instructions given by the physiotherapist. Ethical approval was obtained from the institutional research ethics committee [DMIMS (DU)/IEC/2021/494]. The patients underwent the required assessments and those who fulfilled the inclusion criteria were included in the study. The written informed consent form was taken from patients and the details of the study and outcome measures were explained to them.

NPRS (n=18) and PSS (n=18) scores were measured before and after the intervention.

Statistical analysis

Statistical analysis was done by using descriptive and inferential statistics based on the student’s paired t-tests. All analyses were conducted using the IBM SPSS Statistics version 27.0 (IBM Corp., Armonk, NY). A p-value <0.05 was considered statistically significant.

**Figure 1:**
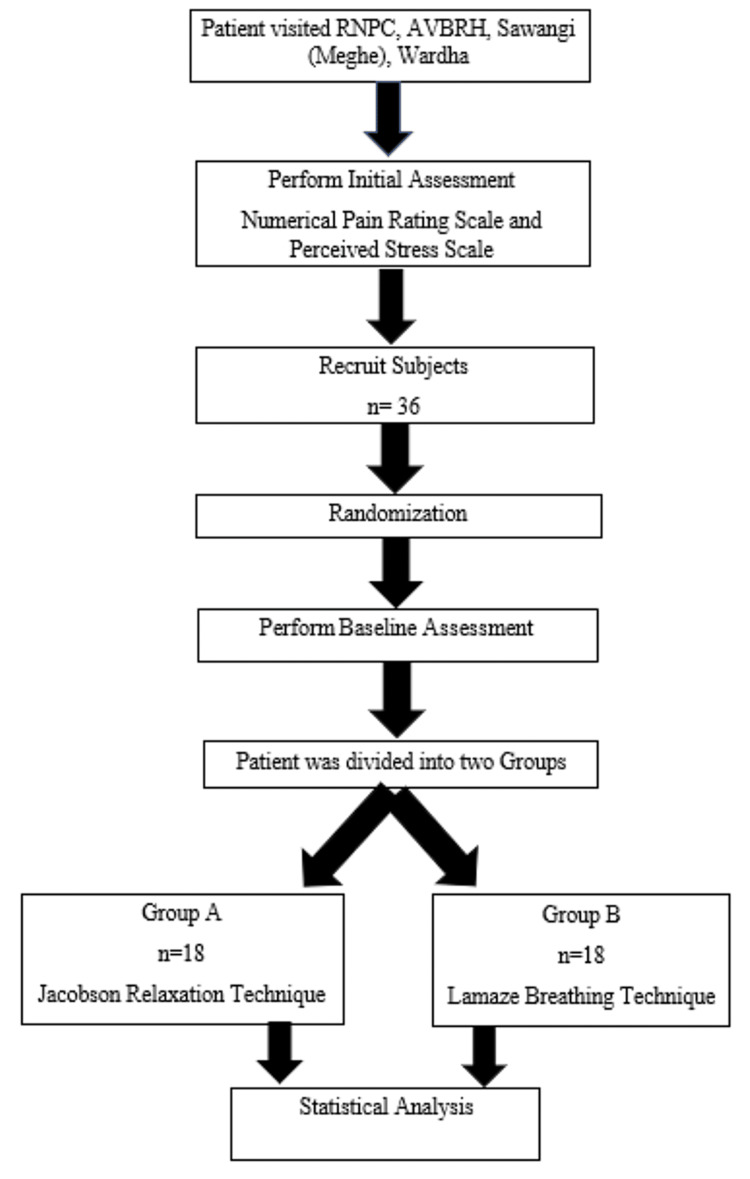
Flow chart of the study process

Figure 1 presents the flow chart depicting the study process.

## Results

**Figure 2:**
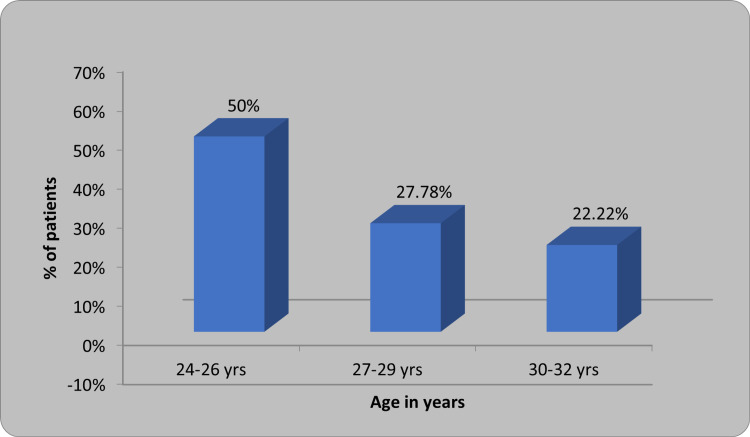
Distribution of patients based on age

As mentioned before, a total of 36 patients were included in the study. Table 1 and Figure 2 present the distribution of patients based on age. The mean ages of women aged 24-26 years, 27-29 years, and 30-32 years were 32 ±16, 18.89 ±8.89, and 15.11 ±7.11 years respectively.

**Table 1:** Distribution of patients based on age

Age in years	Number of patients	Percentage	Mean ±SD
24-26	18	50	32 ±16
27-29	10	27.78	18.89 ±8.89
30-32	8	22.22	15.11 ±7.11
Total	36	100	27.16 ±2.43 (24.32 years)

**Figure 3:**
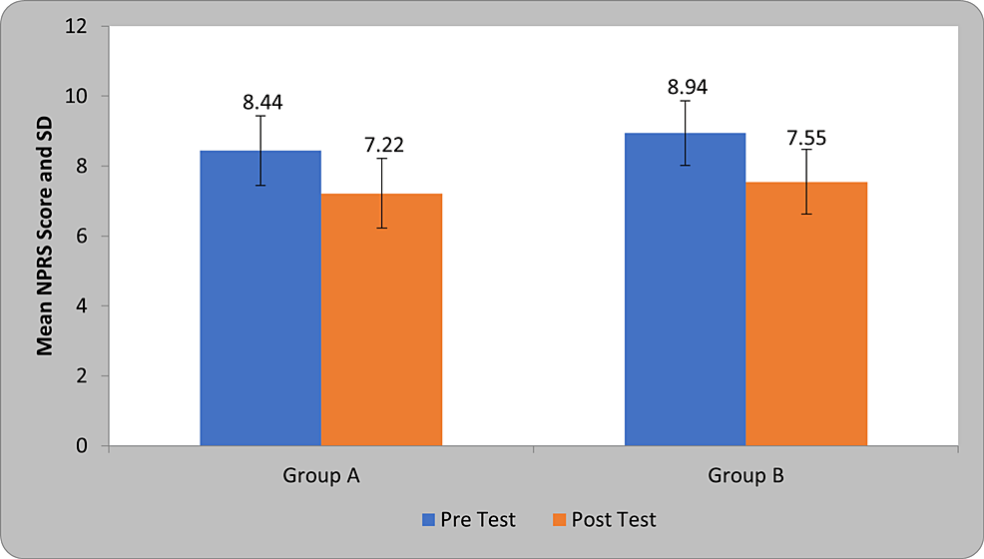
Comparison of Numerical Pain Rating Scale scores between patients of Group A and Group B

The women who underwent the Jacobson relaxation technique were placed in Group A while those who received the Lamaze breathing technique were placed in Group B. The tests employed to check the effectiveness of both techniques were the t-test, NPRS, and PSS. The result of the study highlighted the benefits of the Jacobson relaxation technique and Lamaze breathing. It was observed that the group that was administered Lamaze breathing achieved remarkable results in terms of efficacy on labor pain and stress compared to the group that was administered the Jacobson relaxation technique. The comparison of NPRS scores between patients of Group A and Group B is presented in Table 2 and Figure 3.

**Table 2:** Comparison of Numerical Pain Rating Scale scores between patients of Group A and Group B

Group	Pre-test	Post-test	Mean difference	Student’s paired t-test t-value
Group A	8.77 ±1.00	7.22 ±1.00	1.55 ±0.51	12.90, p=0.0001
Group B	8.94 ±0.93	7.55 ±0.92	1.38 ±0.50	11.74, p=0.0001
Comparison of mean difference in two groups (Student’s unpaired t-test)→			t-value	p-value
			2.434494	0.020023

## Discussion

During labor, pain and stress are the two major issues that affect women. This study aimed to determine how effective the Lamaze breathing method and the Jacobson relaxation technique were in reducing tension and pain during childbirth. The outcome measures used were NPRS and PSS scores of pain and stress during labor. Based on our findings, the Jacobson relaxation technique and Lamaze breathing exercises are very effective in managing labor pain and stress. We found that Lamaze breathing exercises are more effective than the Jacobson relaxation technique.

According to Akköz Çevik and Karaduman, pain and stress are common following labor and hence non-pharmacological treatment is commonly and widely used nowadays as these techniques ensure the relaxation of women without any medications [23]. Woodley et al. have described physiotherapeutic interventions that are used in pregnant women during labor, such as those that alleviate lower back pain and sciatica, and those aimed at pelvic floor strengthening; physiotherapy is beneficial before as well as after the delivery [24]. The study by Hotelling has described the Lamaze breathing technique as a tactic to avoid pain and anxiety during labor as several studies have suggested that Lamaze breathing has been used from ancient periods by midwives, and it involves distracting women from concentrating on labor pain [15]. According to Yeung et al., labor pain is the worst pain experienced by women, and there are many techniques available to treat it, such as the Jacobson relaxation technique and Lamaze breathing exercises, which showed their effectiveness in a randomized control trial. The researchers concluded that these exercises are effective in reducing anxiety during labor pain [25]. A study by Lothian et al. has reported that Lamaze breathing was prevalent in ancient times for preparing a mother for delivery. Labor pain might be a very suffocating experience and Lamaze breathing is taught to women to keep that in check; it also reduces anxiety and perception of pain during labor [6,26]. As per Adler et al., several studies have revealed that cognitive behavioral therapy may also prove effective in reducing anxiety but their study was a trial-based one and the findings have not been validated [27,28].

Lundborg et al. have reported that pharmacological treatment is advised worldwide during labor as this pain is considered the most severe pain anyone can experience. The pain measures may vary from measurable to unbearable, and while a few women can efficiently fight labor pain, most women need pharmacological or physiotherapeutic interventions for relieving pain [29,30]. Physiotherapeutic methods for pain relief and reducing anxiety have been described in various studies. Thomson et al. [31] analyzed trials of these techniques applied during labor and reported that they lead to less pain intensity while increasing the rate of vaginal deliveries without any assistance. In 2016, the World Health Organization highlighted the importance of antenatal guidelines that ensured efficacy, evidence, values, equity, and acceptability for intrapartum care. A qualitative evidence-based study was conducted regarding positive experiences during childbirth, which reviewed the findings of each pain-relieving method and assessed the efficacy of the same. Aya et al. examined the chronobiology of labor pain and reported that hormones such as antinociceptive and peptides are increased during labor leading to pain tolerance in women since these substances have higher diurnal variations with plasma concentration in the morning and could contribute to managing pain perception [32].

Robson et al. [33] stated that cardiac output is essential in the first stage of labor. In their study, cardiac output was assessed by echo color Doppler and echocardiography at the pulmonary valve. During uterine contractions in vaginal delivery, there is an increase in cardiac output as the stroke volume and heart rate also increases. In the advanced stages of labor, the cardiac output progressively increases, and hence breathing exercises are applied to women. These techniques reduce breathlessness and hence heart rate also decreases. O'Driscoll et al. [34] have promoted the active management of labor and stated that this management takes into account the duration of labor pain. The duration of labor can be reduced if every woman is taken care of by the caregivers among hospital staff. This can effectively reduce prolonged labor complications [35].

## Conclusions

Based on our findings, physiotherapy interventions play an integral role in the multidisciplinary approach to pain management during labor. Our results show that physiotherapeutic rehabilitation protocol helps in relaxation and aids in alleviating pregnancy complications that some pregnant females exhibit. It helps patients to relax and improves stress management if followed appropriately. Methods such as the Jacobson relaxation technique and Lamaze breathing exercises can make a world of difference in terms of reducing pain and stress associated with labor.

## References

[1] (2022). Types of pelvis shapes: 4 types and how they affect birth. https://www.healthline.com/health/types-of-pelvis.

[2] (2022). A review and comparison of common maternal positions during the second-stage of labor. https://www.sciencedirect.com/science/article/pii/S2352013219301309.

[3] (2022). What is Jacobson’s relaxation technique?. https://www.healthline.com/health/what-is-jacobson-relaxation-technique.

[4] Smith CA, Levett KM, Collins CT, Armour M, Dahlen HG, Suganuma M (2018). Relaxation techniques for pain management in labour. Cochrane Database Syst Rev.

[5] Progressive Muscle Relaxation: Benefits (2022). The benefits of progressive muscle relaxation and how to do it. https://www.healthline.com/health/progressive-muscle-relaxation.

[6] Lothian JA (2011). Lamaze breathing: what every pregnant woman needs to know. J Perinat Educ.

[7] (2022). Lamaze breathing: how does it work?. https://www.healthline.com/health/lamaze-breathing.

[8] Wang YH, Huang YA, Chen IH, Hou WH, Kang YN (2022). Exercise for trismus prevention in patients with head and neck cancer: a network meta-analysis of randomized controlled trials. Healthcare (Basel).

[9] Petrikovets A, Sheyn D, Sun HH (2019). Multimodal opioid-sparing postoperative pain regimen compared with the standard postoperative pain regimen in vaginal pelvic reconstructive surgery: a multicenter randomized controlled trial. Am J Obstet Gynecol.

[10] Wu C, Ge Y, Zhang X, Du Y, He S, Ji Z, Lang H (2021). The combined effects of Lamaze breathing training and nursing intervention on the delivery in primipara: a PRISMA systematic review meta-analysis. Medicine (Baltimore).

[11] Wu DW, Wang SW, Chang YF, Tsai JH (2020). Effective pharmacotherapy for lung abscess in a patient with alcoholism. Respir Med Case Rep.

[12] Hayes-Skelton SA, Roemer L, Orsillo SM, Borkovec TD (2013). A contemporary view of applied relaxation for generalized anxiety disorder. Cogn Behav Ther.

[13] Ibrahim HA, Elgzar WT, Hablas RM (2021). The effect of Jacobson's progressive relaxation technique on postoperative pain, activity tolerance, and sleeping quality in patients undergoing gynecological surgery. Iran J Nurs Midwifery Res.

[14] Yu H, Higa F, Koide M (2009). Lung abscess caused by Legionella species: implication of the immune status of hosts. Intern Med.

[15] Hotelling BA (2005). Promoting wellness in lamaze classes. J Perinat Educ.

[16] Liu Y, Li T, Guo N, Jiang H, Li Y, Xu C, Yao X (2021). Women's experience and satisfaction with midwife-led maternity care: a cross-sectional survey in China. BMC Pregnancy Childbirth.

[17] Suraci N, Carr C, Peck J, Hoyos J, Rosen G (2020). Improving labour progression among women with epidural anesthesia following use of a birthing ball: a review of recent literature. J Obstet Gynaecol.

[18] Ferendiuk E, Biegańska JM, Kazana P, Pihut M (2019). Progressive muscle relaxation according to Jacobson in treatment of the patients with temporomandibular joint disorders. Folia Med Cracov.

[19] Özlü İ, Öztürk Z, Karaman Özlü Z, Tekin E, Gür A (2021). The effects of progressive muscle relaxation exercises on the anxiety and sleep quality of patients with COVID-19: a randomized controlled study. Perspect Psychiatr Care.

[20] Madden K, Middleton P, Cyna AM, Matthewson M, Jones L (2016). Hypnosis for pain management during labour and childbirth. Cochrane Database Syst Rev.

[21] Williamson A, Hoggart B (2005). Pain: a review of three commonly used pain rating scales. J Clin Nurs.

[22] Baik SH, Fox RS, Mills SD, Roesch SC, Sadler GR, Klonoff EA, Malcarne VL (2019). Reliability and validity of the perceived stress scale-10 in hispanic americans with english or spanish language preference. J Health Psychol.

[23] Akköz Çevik S, Karaduman S (2020). The effect of sacral massage on labor pain and anxiety: a randomized controlled trial. Jpn J Nurs Sci.

[24] Woodley SJ, Boyle R, Cody JD, Mørkved S, Hay-Smith EJ (2017). Pelvic floor muscle training for prevention and treatment of urinary and faecal incontinence in antenatal and postnatal women. Cochrane Database Syst Rev.

[25] Yeung MP, Tsang KW, Yip BH (2019). Birth ball for pregnant women in labour research protocol: a multi-centre randomised controlled trial. BMC Pregnancy Childbirth.

[26] Anim-Somuah M, Smyth RM, Cyna AM, Cuthbert A (2018). Epidural versus non-epidural or no analgesia for pain management in labour. Cochrane Database Syst Rev.

[27] Adler K, Rahkonen L, Kruit H (2020). Maternal childbirth experience in induced and spontaneous labour measured in a visual analog scale and the factors influencing it; a two-year cohort study. BMC Pregnancy Childbirth.

[28] Ranta P, Jouppila P, Spalding M, Jouppila R (1995). The effect of maternal obesity on labour and labour pain. Anaesthesia.

[29] Lundborg L, Liu X, Åberg K, Sandström A, Tilden EL, Stephansson O, Ahlberg M (2021). Association of body mass index and maternal age with first stage duration of labour. Sci Rep.

[30] Abalos E, Oladapo OT, Chamillard M (2018). Duration of spontaneous labour in 'low-risk' women with 'normal' perinatal outcomes: a systematic review. Eur J Obstet Gynecol Reprod Biol.

[31] Thomson G, Feeley C, Moran VH, Downe S, Oladapo OT (2019). Women's experiences of pharmacological and non-pharmacological pain relief methods for labour and childbirth: a qualitative systematic review. Reprod Health.

[32] Aya AG, Vialles N, Mangin R, Robert C, Ferrer JM, Ripart J, de La Coussaye JE (2004). Chronobiology of labour pain perception: an observational study. Br J Anaesth.

[33] Robson SC, Dunlop W, Boys RJ, Hunter S (1987). Cardiac output during labour. Br Med J (Clin Res Ed).

[34] O'Driscoll K, Stronge JM, Minogue M (1973). Active management of labour. Br Med J.

[35] Cavalcanti AC, Henrique AJ, Brasil CM, Gabrielloni MC, Barbieri M (2019). Complementary therapies in labor: randomized clinical trial. Rev Gaucha Enferm.

